# High sensitivity assays for docetaxel and paclitaxel in plasma using solid-phase extraction and high-performance liquid chromatography with UV detection

**DOI:** 10.1186/1472-6904-6-2

**Published:** 2006-01-13

**Authors:** Anders Andersen, David J Warren, Paal F Brunsvig, Steinar Aamdal, Gunnar B Kristensen, Harald Olsen

**Affiliations:** 1 Central Laboratory, The Norwegian Radium Hospital, N-0310 Oslo, Norway; 2 Department of Medical Oncology, The Norwegian Radium Hospital, N-0310 Oslo, Norway; 3 Department of Clinical Research, The Norwegian Radium Hospital, N-0310 Oslo, Norway; 4 Department of Gynecologic Oncology, The Norwegian Radium Hospital, N-0310 Oslo, Norway

## Abstract

**Background:**

The taxanes paclitaxel and docetaxel have traditionally been used in high doses every third week in the treatment of cancer. Lately there has been a trend towards giving weekly low doses to improve the therapeutic index. This article describes the development of high performance liquid chromatographic (HPLC) methods suitable for monitoring taxane levels in patients, focusing on patients receiving low-dose therapy.

**Methods:**

Paclitaxel and docetaxel were extracted from human plasma by solid phase extraction, and detected by absorbance at 227 nm after separation by reversed phase high performance liquid chromatography. The methods were validated and their performance were tested using samples from patients receiving paclitaxel or docetaxel.

**Results:**

The limits of quantitation were 1 nM for docetaxel and 1.2 nM for paclitaxel. For both compounds linearity was confirmed from the limit of quantitation up to 1000 nM in plasma. The recoveries ranged between 92% and 118% for docetaxel and between 76% and 104% for paclitaxel. Accuracy and precision were within international acceptance criteria, that is within ± 15%, except at the limit of quantitation where values within ± 20% are acceptable. Low-dose patients included in an on going clinical trial had a median docetaxel concentration of 2.8 nM at 72 hours post infusion. Patients receiving 100 mg/m^2 ^of paclitaxel had a mean paclitaxel concentration of 21 nM 48 hours after the end of infusion.

**Conclusion:**

We have developed an HPLC method using UV detection capable of quantifying 1 nM of docetaxel in plasma samples. The method should be useful for pharmacokinetic determinations at all relevant doses of docetaxel. Using a similar methodology paclitaxel can be quantified down to a concentration of 1.2 nM in plasma with acceptable accuracy and precision. We further demonstrate that the previously reported negative influence of Cremophor EL on assay performance may be overcome by degradation of the detergent by incubation with lipase.

## Background

**Figure 1:**
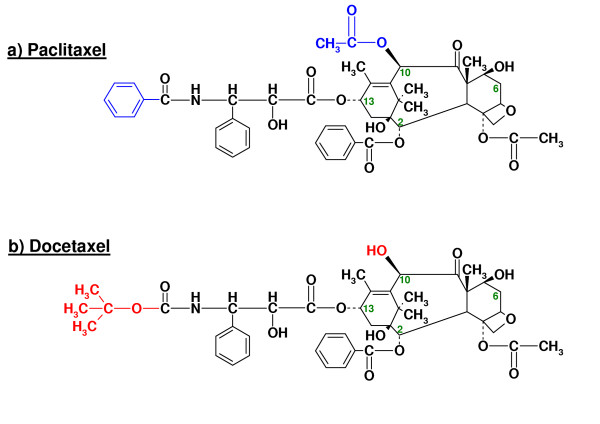
**Molecular structure of paclitaxel and docetaxel**. Molecular structure of paclitaxel (1a) and docetaxel (1b). Dissimilarities are marked in blue (1a) and red (1b).

Paclitaxel (figure 1a) was discovered in the early 1970's as being the active cytotoxic constituent in extracts of the bark of the yew *Taxus Brevifolia*. Paclitaxel had a unique mechanism of action, but the supply was limited and formulation of the compound for clinical use was difficult as paclitaxel is practically insoluble in water. Docetaxel (figure 1b), a semisynthetic analog of paclitaxel with higher aqueous solubility, was constructed from 10-deacetyl baccatin III about 10 years later. Several review articles on the clinical and preclinical pharmacokinetics of paclitaxel and docetaxel have been published [1-3].

Docetaxel for clinical use is currently formulated as a concentrate in polysorbate 80, and about 4 ml of this non-ionic detergent is infused together with a standard dose (173 mg) of docetaxel. The plasma concentration of polysorbate 80 is about 0.1% during the infusion and the compound is rapidly cleared from plasma [4,5]. This is in contrast to Cremophor EL, the solvent for paclitaxel, where about 27 ml is infused together with a standard dose (303 mg), and the plasma concentration remains high (above 0.1%) for days due to a low volume of distribution (3 L/m^2^) and a long half-life (40–80 h) [6-10]. Cremophor EL has been reported to have a negative influence on the reproducibility of paclitaxel determinations [11]. No such effects have been reported for polysorbate 80 and docetaxel.

Numerous analytical methods for the taxanes have been published. Most of them rely on sample preparation by solid phase extraction followed by separation by HPLC and detection by absorbance at 225–230 nm. The majority of methods for both paclitaxel and docetaxel are based on the principles for extraction and separation published by Willey et al. [12] in 1993, but also methods based on organic extraction are used [13-15]. Lately, mass spectrometry (MS) based methods with simplified sample preparations and limits of detections in the sub-nanomolar range have appeared [4,16-19]. The improved sensitivity has given new insight into the pharmacokinetics of the taxanes [20,21].

The method development presented here was started to support a dose escalation study with paclitaxel in patients with ovarian cancer. As we lacked an MS-instrument, the detection utilized UV absorbance. Later on the development of an HPLC method for docetaxel was initiated, based on the experiments performed with paclitaxel.

We present here a validated reversed phase HPLC method based on UV detection that has sufficient sensitivity to perform pharmacokinetic determinations at all relevant doses of docetaxel. With a similar methodology, paclitaxel may be quantified down to a concentration of 1.2 nM in plasma. We also demonstrate that it is possible to eliminate Cremophor EL from plasma samples by degradation of the detergent with lipase.

## Methods

### Chemicals and reagents

Docetaxel reference standard (lot 9915420, purity 98.5%) was initially supplied by Aventis Pharma (Vitry Alforville research Center, France) but later purchased from ARC Inc. (St. Louis, MO, USA). Cremophor EL, ^14^C-paclitaxel ([2-benzoyl ring-UL-^14^C], 62.9 mCi/mmol in ethyl acetate) and semisynthetic paclitaxel was purchased from Sigma Chemical Co. (St. Louis, MO, USA). ^3^H-paclitaxel (10.5 Ci/mmol in ethanol) and natural non-labelled paclitaxel was purchased from Moravek Biochemicals (Brea, CA, USA). 2'-methylpaclitaxel (lot 001, purity 95%) was obtained from Bristol-Meyers Squibb (Syracuse, NY, USA). Coomassie Brilliant Blue G-250 was purchased as a concentrate from Bio-Rad Laboratories (Munich, Germany). Lipase from Rhizopus arrhizus (EC 3.1.1.3), 50 kU/ml, was purchased from Roche (Basel, Switzerland). HPLC grade methanol was from Mallinckrodt Baker B.V. (Deventer, Holland). Acetonitrile (HPLC grade) and sequencer grade n-butyl chloride were from Rathburn (Walkerburn, UK). FluoranFlow scintillation fluid was from BDH (Poole, UK). Drug-free EDTA plasma was obtained from healthy donors.

### Preparation of standards

Stock solutions of taxane reference standards were made up in acetonitrile and stored at -70°C. Further dilutions to make calibration standards in EDTA plasma were performed on the day of use. ^3^H-paclitaxel was used as internal standard for the determination of paclitaxel. The received standard was diluted 1:20 in acetonitrile, aliquoted in glass vials and stored at -70°C. Before use these solutions were further diluted in acetonitrile to a final concentration of 0.45 nCi/μl, and 20 μl were added to each 4 ml sample of plasma giving a final paclitaxel concentration of 0.2 nM. ^14^C-paclitaxel was used as a tracer during the methods development. The received concentrate was diluted in ethanol and stored at -70°C in glass vials.

### Instrumentation

All chromatographic equipment was produced by Shimadzu Corp. (Tokyo, Japan). The solvent delivery system consisted of a LC-9A quartenary gradient pump, a DGU-3A on-line degasser and a CTO-6A column oven and on-line solvent preheater. Samples were injected with a SIL-6B autoinjector and detected by a SPD-6AV variable wavelength UV detector. Data acquisition and integration were performed by a Class-VP 4.2 computer-based integration system. Quantitations of ^14^C-labelled and ^3^H-labelled paclitaxel were undertaken using a 1211 RackBeta liquid scintillation counter from Wallac Oy (Turku, Finland).

### Sample processing

The taxanes were extracted from samples of human plasma using solid phase extraction (SPE). The individual steps in the sample preparation are tabulated in table 1, together with the two methods we used as basis for this method development. SPE were performed using columns containing 100 mg of CN packing material (with or without endcapping, see table 1) and having a reservoir capacity of 10 ml (LRC-SPE columns, Varian, Harbor City, CA, USA).

**Table 1:** Solid Phase Extraction

	**Paclitaxel**		**Docetaxel**	
**Author**	**Willey [12]**	**Andersen et al**	**Rosing [24]**	**Andersen et al**

**Columns**	1 ml CN-(?) 100 mg^#^	LRC CN-U 100 mg	CN-E 100 mg	LRC CN-E 100 mg
**Cond 1**	2 ml MeOH	10 ml MeOH	1 ml 0.1% TEA in MeCN	10 ml MeOH
**Cond 2**	2 ml AmAc^##^	5 ml AmAc	2 ml MeOH	2 ml AmAc
**Cond 3**	None	None	2 ml AmAc	None
**Load**	1 ml 50% plasma*	Up to 8 ml 50% plasma*	1.1 ml undiluted plasma	2 ml undiluted plasma
**Wash 1**	2 ml AmAc	10 ml AmAc	2 ml AmAc	2 ml AmAc
**Wash 2**	2 ml AmAc/20% MeOH	1 ml AmAc/20% MeOH	1 ml water/20% MeOH	1 ml AmAc/20% MeOH
**Wash 3**	1 ml hexane	5 ml hexane	None	2 ml hexane
**Elution**	2× 1 ml 0.1% TEA in MeCN	2× 250 μl ethyl acetate	1× 500 μl 0.1% TEA in MeCN	2× 250 μl ethyl acetate
**Evap**	Nitrogen gas blowdown	Vacuum centrifuge	Nitrogen gas blowdown	Vacuum centrifuge
**Injection volume**	100 μl (of 200 μl) in MeOH/MeCN/AmAc	90 μl (of 100 μl) in 40% MeCN	50 μl (of 100 μl) in MeOH/MeCN/water	90 μl (of 100 μl) in 40% MeCN

The taxanes were extracted from samples of human plasma using solid phase extraction. The individual steps in the solid phase extraction are shown, together with the two methods we used as basis for this method development.

MeOH = methanol, MeCN = acetonitrile

# Not specified if the columns were endcapped or not.

## AmAc = 10 mM ammonium acetate buffer pH 5.0.

* The plasma samples were mixed with an equal volume of 0.2 M ammonium acetate pH 5.0.

The activation, sample extraction and the first two washes were performed by gravity, i.e. no vacuum was used. Twenty microlitres of internal standard was added to the plasma samples: for the determination of paclitaxel ^3^H-paclitaxel (0.45 nCi/μl), for the determination of docetaxel 20 μM semisynthetic paclitaxel. The plasma samples were mixed thoroughly and centrifuged at 2000 g for 15 minutes. The full volume of sample, leaving only the pellet was pipetted onto the activated SPE-columns. After the second wash, vacuum was applied and the columns were washed with hexane and dried by running air through the columns at full vacuum for 3 minutes. The taxanes were eluted into 1.1 ml tapered glass vials (Chromacol 1.1-STVG) with two successive 250 μl aliquots of ethyl acetate. The samples were evaporated to dryness in a vacuum centrifuge and dissolved in 40% acetonitrile by sonication for 2 minutes in a Branson 1200 ultrasonic waterbath followed by vortex mixing. Ninety microlitres of sample was injected onto the HPLC system.

### HPLC conditions

The paclitaxel samples were separated on a Supelcosil LC-18 column (4.6 × 150 mm, particle size 3 μm; Supelco Inc, PA, USA) protected by a 0.5 μm precolumn filter and a 20 mm LC-18 Supelguard. The mobile phase consisted of 20 mM potassium phosphate buffer (pH 3.0): acetonitrile (55:45 v/v) delivered at a flow rate of 1 ml/min. The buffer was made up by pH adjustment of a solution of phosphoric acid with 10 M potassium hydroxide. The column temperature was maintained at 40°C, and the UV detector was operated at 227 nm.

The docetaxel samples were separated on a Purospher^® ^STAR RP-18e column (3.0 × 125 mm, particle size 3 μm; Merck, Darmstadt, Germany) protected by a 0.5 μm precolumn filter and a 4 mm guard cartridge. The mobile phase consisted of 20 mM potassium phosphate buffer (pH 3.0): acetonitrile (57.5:42.5 v/v) delivered at a flow rate of 0.8 ml/min. The column temperature was maintained at 55°C, and the UV detector was operated at 227 nm.

### Determination of Cremophor EL

With some minor modifications, Cremophor EL concentrations in plasma were determined by the linearized version [8] of the method of Sparreboom et al. [22]. Twenty-five microlitres of plasma was deproteinized by the addition of 250 μl acetonitrile in 2 ml micro centrifuge tubes (Costar 3213) followed by vortex mixing. One ml of n-butyl chloride was added, and after a brief mixing the tubes were placed in a sonicating waterbath at 40°C for 10–15 minutes. The organic layer was separated by centrifugation at 26000 g for 5 minutes in a Hermle Z252MK centrifuge (Maschinenfabrik Berthold Hermle AG, Gosheim, Germany), transferred to a clean 2 ml micro centrifuge tube, and evaporated to dryness in a vacuum centrifuge. The residue was reconstituted in 100 μl of 10% methanol in water (v/v), and duplicate volumes of 40 μl were transferred to flat bottom 96-well micro titre plates (Nunc A/S, Roskilde, Denmark). Two hundred and fifty microlitres of diluted (1:10 in water v/v) Coomassie Brilliant Blue concentrate was added to each well, and the absorbance at 620 and 450 nm were measured with a 1420 Victor multilabel counter (Wallac Oy, Turku, Finland). Cremophor EL concentrations in plasma were calculated from the absorbances as outlined by Brouwer [8].

### Validation

The analytical methods were validated regarding linearity, recovery, accuracy, precision, sensitivity and sample volume based on the criteria described by Shah et al. [23].

#### Solid phase extraction, recovery studies

Total recovery was determined by comparing peak areas of processed samples with peak areas of standards in 40% acetonitrile injected directly without processing. To facilitate the recovery-studies, ^14^C-paclitaxel was used as tracer, as samples of flow-through and wash solutions from the SPE-procedure could not be injected directly onto the HPLC. Recovery studies of every step in the extraction procedure of Willey et al. [12] were performed to improve the sensitivity of the assay.

To determine maximum sample capacity and losses during the sample preparation, three samples of 5 ml EDTA-plasma containing 50 nM ^14^C-paclitaxel were mixed with an equal volume of a 200 mM ammonium acetate buffer pH 5.0 and loaded by gravity onto activated SPE-columns. Except for the increased sample volume, the SPE-procedure was performed as outlined by Willey et al. [12]. The flow-through, washes and eluates were collected in fractions, and paclitaxel concentration determined by scintillation counting.

To determine the optimal flow during the extraction, a total of 18 samples of 4 ml EDTA-plasma containing 500 nM paclitaxel (^14^C-paclitaxel as tracer) were mixed with an equal volume of a 200 mM ammonium acetate buffer pH 5.0 and extracted with a flow ranging from 0.1 to 7 ml/min. Paclitaxel concentration in the eluates were determined by scintillation counting.

Recovery studies of each step in the extraction process were not performed with docetaxel, as radiolabelled docetaxel was not available. For this compound, the total recovery was used to evaluate the effects of the modifications made to the extraction method.

#### Linearity, accuracy and precision

For paclitaxel, the linearity was determined by analysing calibration standards in duplicate at five levels (1000, 100, 10, 1.2 and 0.7 nM), and for docetaxel at eight levels (1000, 500, 250, 100, 25, 10, 2.5 and 1 nM) using single samples. The validation for both compounds included five calibrator curves analysed over a period of 8–10 days. Three quality control (QC) samples at each level were prepared separately and used for determining accuracy and precision for the paclitaxel method. In the analysis of docetaxel, QC samples in triplicate at three levels (750, 50 and 5 nM) were included in each run and used for determination of accuracy and precision. Additional samples (5 at each level) containing 1 nM and 2 nM of docetaxel were included to verify the limit of quantitation. All calibrator curves were calculated using weighted least squares regression (WLS) using a weighing factor of 1/x, where x is the analyte concentration.

#### Influence of Cremophor EL on assay performance

Eighteen samples of 4 ml EDTA-plasma containing 0.5% Cremophor EL and eighteen samples containing 1% Cremophor EL were prepared. Nine samples with Cremophor EL-free plasma served as controls. All samples contained 50 nM of paclitaxel with ^14^C-paclitaxel as tracer. Lipase was added to an enzyme concentration of 125 U/ml plasma to 9 samples in each group. The samples were incubated at 37°C for 30 min to enzymatically degrade the Cremophor EL. The Cremophor EL concentrations were determined after 0, 2, 5, 10, 15 and 30 minutes of incubation. Subsequent to enzyme treatment, the samples from the 1%-group were extracted in triplicate on three different batches of SPE-columns together with the Cremophor EL-free samples. Paclitaxel concentrations in the eluates were determined by scintillation counting.

### Patients

Blood samples were obtained from 10 patients receiving 175 mg/m^2 ^of paclitaxel as a 3 hours infusion as a part of the treatment for advanced ovarian carcinoma. The samples were collected in EDTA tubes prior to the infusion, and at 2 hours, 24 hours and 48 hours after the end of the infusion. Plasma was frozen at -70°C until analysis. All patients gave written informed consent.

In the docetaxel study, blood samples were obtained from 5 patients. One patient receiving 20 mg/m^2 ^of docetaxel and four patients receiving 100 mg/m^2 ^of docetaxel as a 1-hour infusion as a part of the treatment for non-small cell lung cancer. The samples were collected in EDTA tubes prior to the infusion, and at 1 hour, 2 hours and 24 hours after the end of the infusion. Samples were collected from two consecutive treatments in each patient. Plasma was frozen at -70°C until analysis. All patients were enrolled in clinical trials approved by the Regional Committee for Medical Research Ethics and the Hospital Review Board. The studies were performed in compliance with the World Medical Association Declaration of Helsinki. Written informed consent was obtained from all patients.

## Results and discussion

### Solid Phase Extraction, recovery studies

Recovery studies with paclitaxel using high sample volumes (10 ml of diluted plasma) by the method of Willey et al. [12] showed that losses during loading were 2% for the first 4 ml of sample, 2.5% for the next 3 ml, and 4% for the final 3 ml. A loss of 1% was observed in the first wash with 2 ml acetate buffer. Another 3% was lost in the second wash with 2 ml 20% methanol in acetate buffer. Only 0.2% was lost during the wash with hexane. An average of 86% of the loaded radioactivity was recovered in the eluate.

**Figure 2:**
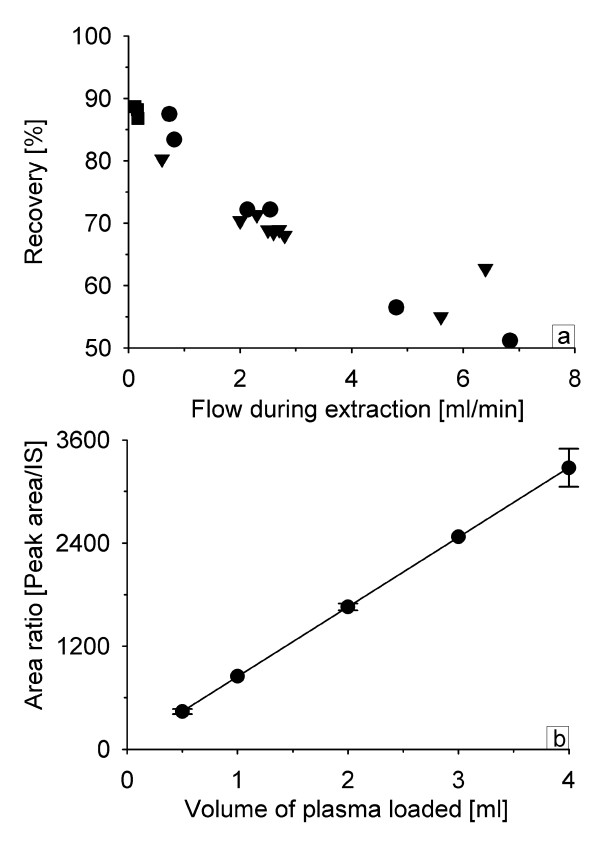
**Influence of sample flow and sample volume on paclitaxel recovery**. **2a: Influence of the flow during the extraction on paclitaxel recovery**. A total of 18 samples of 4 ml EDTA-plasma containing ^14^C-paclitaxel as tracer were extracted with a flow ranging from 0.1 to 7 ml/min in 3 separate experiments (experiment 1: ●, 2: ▼ and 3: ■). **2b: Relationship between sample size and response**. Using a sample size of 0.5, 1, 2, 3 and 4 ml of plasma with a flow of <0.5 ml/min during the extraction caused a linear increase in detector response with increasing sample size. Data from 3 separate experiments with a total number of 25 determinations are included in figure 2b. The data points given are mean values with error bars representing ± 1SD.

Increasing the flow during the extraction from 0.1 to 7 ml/min had a strong negative effect on recovery (figure 2a). Similarly, Rosing [24] found that 0.4 ml/min was optimal in their automated docetaxel extraction. Obtaining a low (<1 ml/min) and reproducible flow rate is difficult using vacuum. Therefore all subsequent experiments were performed by gravity (no vacuum) during conditioning, loading of sample, and the first two washes. Extracting by gravity also enabled one person to process many samples in parallel as the columns do not dry out, the flow will stop as the liquid reaches the top frit.

After determining the optimal conditions for loading of large sample volumes, the next step was to improve the washings to obtain clean extracts from large samples. The volume of the first wash was increased from 2 ml to 10 ml, the volume of the second wash was reduced from 2 ml to 1 ml as recommended by Huizing et al. [11], and the volume of the hexane-wash was increased to 5 ml.

Even with these modifications we still observed a number of background/endogenous peaks in the chromatogram, and when extracting from large sample volumes particles in the reconstituted samples. As some of the interfering peaks were present also in reagent blanks, i.e. samples without plasma, all reagents were checked separately and we found that several of the peaks originated from the elution of samples into polypropylene tubes.

A screening of solvents suitable for eluting paclitaxel showed that non-water miscible organic solvents gave the cleanest extracts. Ethyl acetate eluted 89 ± 2% (mean ± 1SD) of paclitaxel in the first 250 μl, 3 ± 1% in the second and 0.3 ± 0.1% in the third aliquot of 250 μl. Using only 2 aliquots of 250 μl of ethyl acetate, a near quantitative elution was obtained. The low volume of solvent enabled elution directly into 1.1 ml glass injection vials, and the solvent was completely evaporated within 60 minutes in a vacuum centrifuge. Reconstitution in 40% acetonitrile gave particle-free samples that could be injected without prior centrifugation. Losses due to sample transfer were thereby minimized. With these modifications, and using ^3^H-paclitaxel as internal standard, samples of 0.5, 1, 2, 3 and 4 ml of plasma were extracted, and gave a linear increase in response with increasing sample size (figure 2b).

Attempts to use the paclitaxel extraction method also for docetaxel were unsuccessful due to low and variable recovery. Rosing et al. [24] showed that end-capped cyano columns were more suitable for the extraction of docetaxel from plasma. This method however, did not have sufficient sensitivity for undertaking pharmacokinetic determinations during low-dose therapy. Using the experiences learned from the development of the method for paclitaxel, we performed similar modifications to Rosings method attempting to obtain a lower limit of detection. The sample volume was increased to 2 ml, and the washings and elution was modified (table 1). Plastic tubes were used for storing plasma samples, but not for reagents, standards in acetonitrile or eluates in ethyl acetate.

### Chromatography

We used columns with 3 μm particles instead of 5 μm particles for both methods. The smaller particles provide a higher number of theoretical plates, and thereby sharper chromatographic peaks and a better signal to noise ratio. This may lower the limit of detection as reproducible peak integration is facilitated.

The mobile phase used in the original method (acetate-buffer/MeCN/MeOH) was found to have a substantial absorbance at 227 nm, and a long equilibration was necessary to obtain a stable baseline. To lower the absorbance, methanol was omitted in the mobile phase and acetonitrile was used to adjust retention. The taxanes used in this method did not have any ionizable groups in the pH-range used for HPLC. The acetate buffer in the mobile phase could be substituted for a 20 mM potassium phosphate buffer pH 3.0 without affecting retention and with a favorable effect on mobile phase absorbance.

Quantifying docetaxel in plasma by UV-detection is a challenging task as docetaxel has a lower absorbance than paclitaxel and is used in lower doses. To increase the signal height, and thereby the sensitivity, the 4.6 mm ID column used in the paclitaxel method was replaced by a 3 mm ID column in the docetaxel method. The nominal increase in signal height is 2.4 times when the ID of the column is reduced from 4.6 to 3 mm. The acetonitrile content of the mobile phase and the temperature were adjusted to optimize the separation between docetaxel and the internal standard.

Paclitaxel of natural origin could not be used as internal standard due to impurities interfering with the docetaxel peak. Another candidate used by others, 2-methyl-paclitaxel, also contained impurities and was excluded due to late eluters. Semisynthetic paclitaxel (Sigma T 7191) had sufficient purity to be suitable as an internal standard for the docetaxel method.

### Influence of Cremophor EL on assay performance

**Figure 3:**
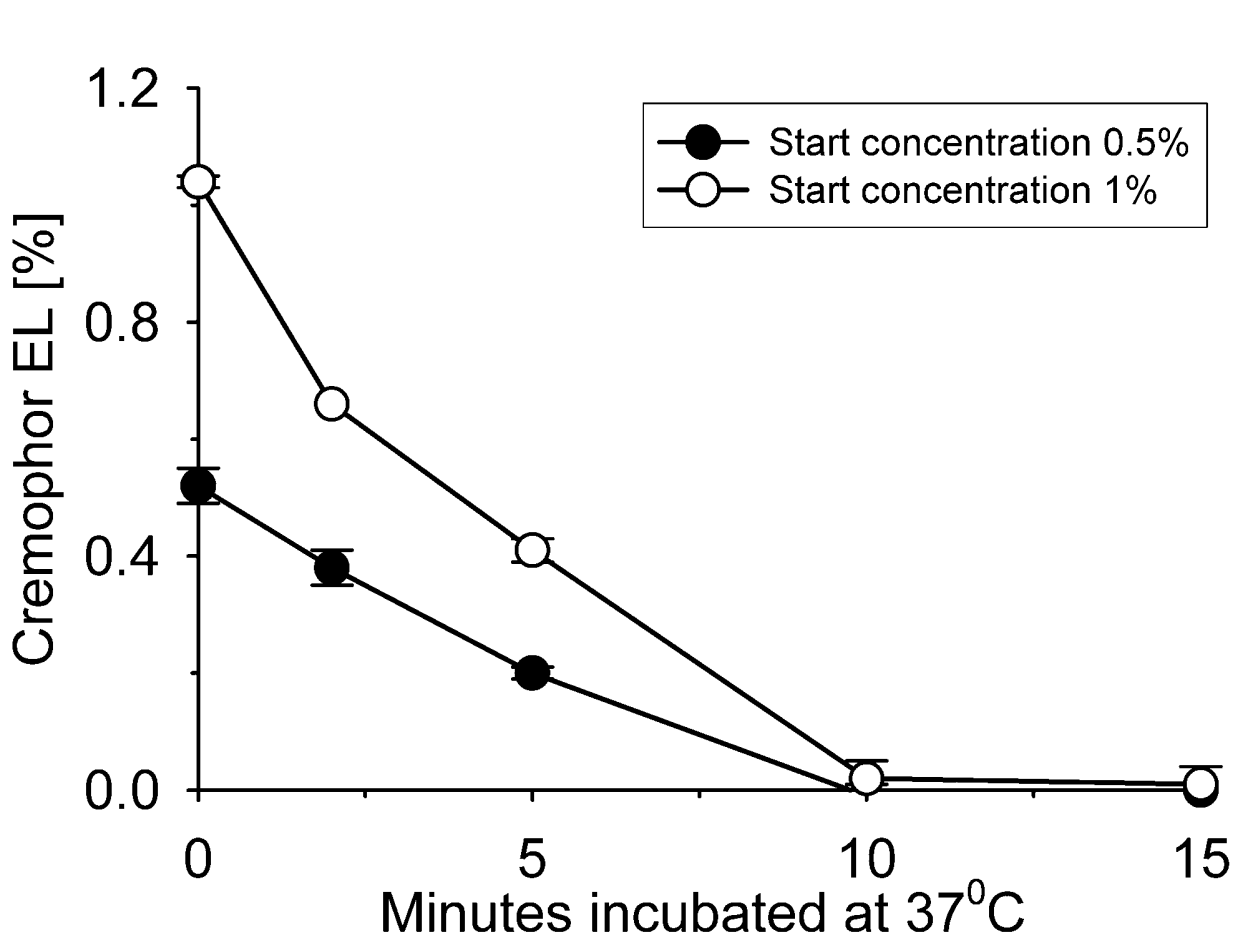
**Degradation of Cremophor EL with lipase**. Samples of EDTA-plasma containing 0.5% and 1% Cremophor EL were incubated at 37°C with 125 units of lipase/ml plasma. The data points given are mean values with error bars representing ± 1SD.

**Figure 4:**
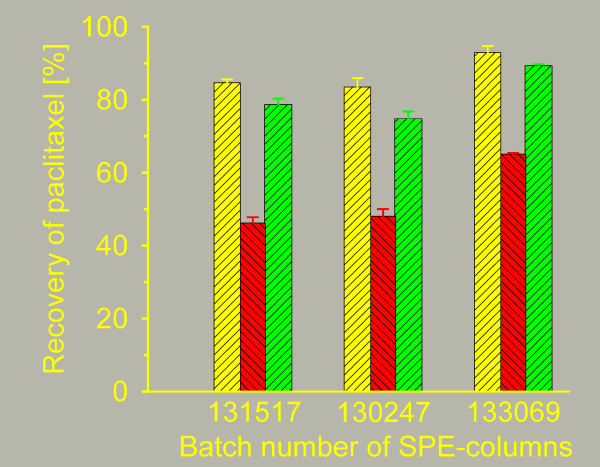
**Influence of Cremophor EL on extraction performance**. Recovery of paclitaxel from plasma samples containing 1% Cremophor EL before () and after () lipase treatment, compared to plasma samples without Cremophor EL (). Three different batches of extraction columns were tested. The bars shown are mean values with error bars representing ± 1SD.

The experiments showed that Cremophor EL in plasma could be rapidly degraded by incubation with lipase. After 10 minutes of incubation the concentration of Cremophor EL was near the limit of detection for the method (figure 3). Solid phase extraction of the samples from the 1%-group confirmed the results of Huizing et al. [11] regarding the negative effect of Cremophor EL on the performance of the extraction method, but also revealed that lipase treatment of the samples diminish the negative effect (figure 4).

### Analytical performance

Because of the wide concentration range of the calibrator curves for both paclitaxel and docetaxel, using WLS regression instead of classical linear regression is recommended to prevent minor errors in the high end of the calibrator curves resulting in major errors in the quantification of samples in the low end of the calibrator curves [25,26].

#### Paclitaxel

Using a sample size of four milliliters of plasma, the relationship between the drug concentration and the area ratio was linear from 1.2 to 1000 nM paclitaxel in plasma. The lowest level tested, 0.7 nM, was not included in the calibrator curves due to deviation from linearity. WLS regression of five calibrator curves in duplicate gave the linear equation: Amount = 0.148(± 0.002) × Ratio – 0.149(± 0.2), r^2 ^ranged between 0.998 and 1.000.

Inter- and intra-assay accuracy and precision are presented in table 2. Based on these results the limit of quantitation was set at 1.2 nM of paclitaxel. Recovery ranged between 76 and 104% for the QC samples.

**Table 2:** Intra- and inter-assay accuracy and precision (CV%) for paclitaxel and docetaxel determinations in plasma.

	Concentration [nM]	Intra-assay variability			Inter-assay variability		
			
		n	Accuracy (%)	CV (%)	n	Accuracy (%)	CV (%)
Paclitaxel	1000	3	89	2	5	100	2
	100	3	103	6	5	103	3
	10	3	111	2	5	104	9
	1.2	3	104	8	5	108	14
	0.7	3	105	20		ND	
Docetaxel	750	3	100	1	5	101	3
	50	3	97	1	5	94	6
	5	3	99	4	5	100	5
	2	5	93	4		ND	
	1	5	96	12		ND	

ND = Not done.

#### Docetaxel

For the docetaxel assay, we used a sample size of two milliliters of undiluted plasma. The method was linear over the range tested, from 1 nM to 1000 nM docetaxel in plasma. WLS regression of five calibrator curves with calibration standards at eight levels gave the linear equation: Amount = 277(± 6) × Ratio – 0.6(± 0.3), r^2 ^ranged between 0.999 and 1.000.

QC samples were run at three levels, 750 nM, 50 nM and 5 nM docetaxel in plasma. Inter- and intra-assay accuracy and precision are presented in table 2. Recovery for the standards from 1000 nM to 2.5 nM was 92 ± 5%, for the 1 nM level the recovery was 118 ± 11%. Based on these results, the limit of quantitation was set at 1 nM of docetaxel. Results from the additional samples containing 1 nM and 2 nM of docetaxel, included to verify the limit of quantitation, may be found in table 2.

### Patients

#### Paclitaxel

**Figure 5:**
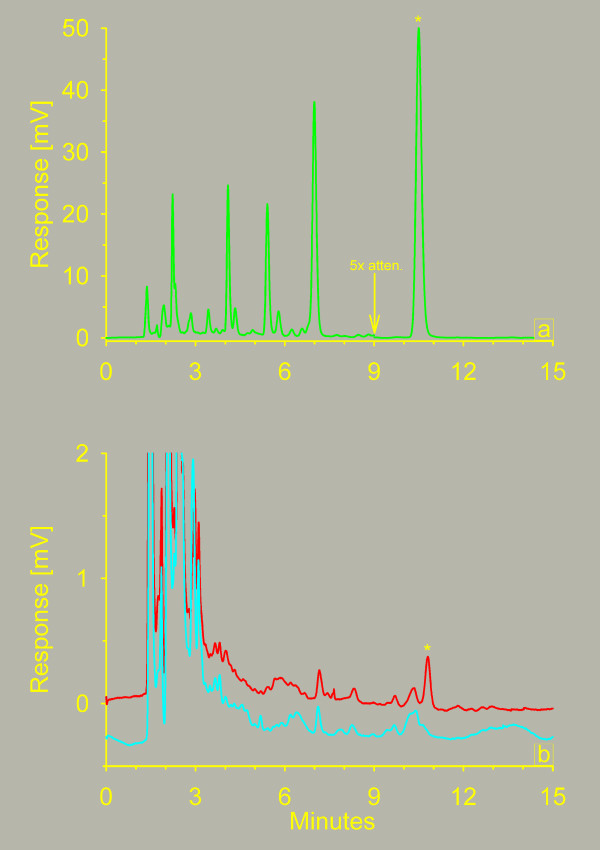
**Chromatograms of plasma samples containing paclitaxel**. 5a: Chromatogram from a patient sample taken 2 hours after the infusion of 175 mg of paclitaxel/m^2 ^to a patient. The paclitaxel peak is marked by an asterisk (). The signal is attenuated by a factor of 5 from nine minutes to get all peaks on scale. 5b: Chromatograms of a calibration sample containing 1.2 nM of paclitaxel () and a plasma blank (). The paclitaxel peak is marked by an asterisk ().

Chromatograms from the analysis of patient samples and standards are shown in figure 5. At 2, 24, and 48 hours after the end of infusion, the paclitaxel plasma concentrations were 860 ± 240 nM, 57 ± 16 nM and 21 ± 7 nM respectively.

#### Docetaxel

**Figure 6:**
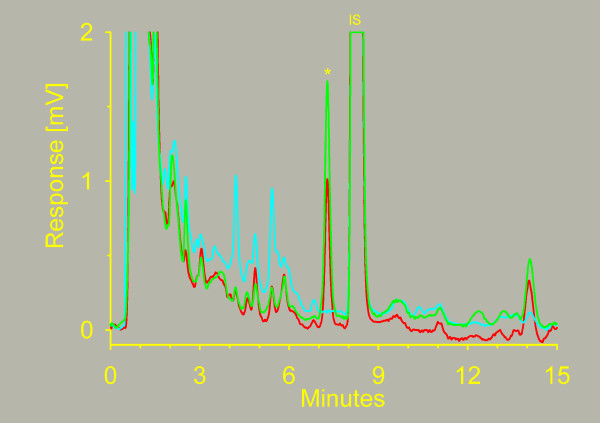
**Chromatograms of plasma samples containing docetaxel**. Chromatograms of blank plasma (), a calibration sample containing 5 nM of docetaxel () and a patient sample containing 8.5 nM of docetaxel (). The docetaxel peak is marked by an asterisk (). The internal standard peak is marked ().

Chromatograms from the analysis of patient samples and standards are shown in figure 6. At 1, 2, and 24 hours after the end of infusion, the docetaxel concentration in the high-dose patients were 370 ± 140 nM, 170 ± 50 nM and 23 ± 3 nM respectively, and 100 nM, 60 nM and 8.1 nM in the low-dose patient. Seeing that the limit of quantitation was as low as 1 nM of docetaxel, it was decided to increase the sampling period in the clinical study to 72 hours to get a more complete picture of the elimination of docetaxel. Median docetaxel concentration at 72 hours in the low-dose patients included so far was 2.8 nM.

### Stability

Stability of paclitaxel and docetaxel has been studied extensively by others. Paclitaxel appeared to be stable in frozen (-20°C) human plasma for up to two years, and up to three freeze-thaw cycles did not affect recovery. Refrigerated at +4°C, paclitaxel was stable for at least 24 hours in human plasma. At room temperature however, storage should be limited to 4–8 hours [12,17]. Samples reconstituted in 40% MeCN were stable for at least 24 hours [27]. Similar results have been found regarding stability of docetaxel [4,19,24]

## Conclusion

We have developed an HPLC method using UV detection capable of quantifying 1 nM of docetaxel in plasma samples, which is sufficient sensitivity to undertake determinations up to 72 hours after an infusion of 20 mg/m^2 ^of docetaxel. Cancer cell lines tested *in vitro *gave IC_50_-values for docetaxel ranging from 5 to 40 nM [2]. Using this range as a guideline for which blood concentrations of docetaxel may be considered as pharmacologically active, the method presented here should be useful for pharmacokinetic determinations at all relevant doses of docetaxel.

Using a similar methodology paclitaxel can be quantified down to a concentration of 1.2 nM in plasma with acceptable accuracy and precision. If we were to use the paclitaxel method in large series of samples however, we would consider testing out two modifications: first finding a more convenient internal standard than ^3^H-paclitaxel, and second to reduce the sample size to 2 ml plasma and maintaining the sensitivity by reducing the column internal diameter to 3 mm.

Chromatographic methods based on UV detection will inevitable involve a more complex sample preparation than methods based on MS detection. This should however be weighted against the costs of purchasing and running LC-MS systems.

## Competing interests

The author(s) declare that they have no competing interests.

## Authors' contributions

AA was the main designer, performed the laboratory work presented in this paper and drafted the manuscript. DJW and HO participated in designing the study, and in manuscript preparation. PFB and SAa participated in designing the study and in the selection of patients. GBK participated in the selection of patients. All authors read and approved the final manuscript.

## Pre-publication history

The pre-publication history for this paper can be accessed here:


